# Establishment of Uveal Melanoma Patient-Derived Organoids Using an Air–Liquid Interface System for Translational Research

**DOI:** 10.1167/iovs.67.12.1

**Published:** 2026-10-01

**Authors:** Leticia Ussem, Karin U. Loeffler, Verena Tischler, Marieta Toma, Natalie Pelusi, Frank G. Holz, Anne Gulbins, Daniela Süsskind, Roberta Noberini, Tiziana Bonaldi, Martina C. Herwig-Carl

**Affiliations:** 1 University of Bonn, University Hospital Bonn, Department of Ophthalmology, Bonn, Germany; 2 Division of Ophthalmic Pathology, University Hospital Bonn, Bonn, Germany; 3 Mildred Scheel School of Oncology Aachen Bonn Cologne Düsseldorf (MSSO ABCD), Bonn, Germany; 4 Institute of Pathology, University Hospital Bonn, Bonn, Germany; 5 Centrum for Integrated Oncology Aachen Bonn Cologne Düsseldorf (CIO ABCD), Germany; 6 Department of Ophthalmology, University Hospital Tübingen, Tübingen, Germany; 7 Department of Experimental Oncology, European Institute of Oncology (IEO) IRCCS, Milan, Italy; 8 Department of Oncology and Haemato-Oncology, University of Milan, Milan, Italy

**Keywords:** PDO, epigenetics, histology, HDACi, uveal melanoma

## Abstract

**Purpose:**

Uveal melanoma (UM) is the most common primary intraocular malignancy in adults, with a unique genetic and epigenetic profile and a high metastatic risk. The lack of experimental models to replicate tumor biology, including its epigenetic landscape, limits progress in translational research. Recently, patient-derived organoid (PDO) models have emerged as a promising tool for the modeling of various tumors, including UM. This study aims to establish an air–liquid interface (ALI) PDO culture system for UM, evaluate its similarity to the primary tumors, and assess its utility for drug screening.

**Methods:**

Fresh and cryopreserved tissue samples from primary UM of 5 patients were cultured using a protocol previously validated for other malignancies. The subsequent characterization of the ALI PDOs included histologic and immunohistochemical evaluation, DNA and RNA sequencing, and histone posttranslational modification (HPTM) analysis. The model's drug-screening potential was evaluated by treating ALI PDOs with the histone deacetylase inhibitor quisinostat and comparing their responses with those of controls.

**Results:**

UM ALI PDOs were successfully established and retained their original tumors’ immunophenotype and genetic variants. Gene expression profiling revealed similarities between PDOs and primary tumors, with main differences consisting of microenvironment-related signals. Epigenetic analysis confirmed that the overall HPTM landscape remained preserved. Treatment of the PDOs with quisinostat led to transcriptional reprogramming and the expected epigenetic response, with increased acetylation marks.

**Conclusions:**

The UM ALI PDO model recapitulates the genetic and epigenetic features of primary UM and holds promise as a platform for future molecular studies and the testing of personalized therapy strategies.

Uveal melanoma (UM) is the most common primary intraocular neoplasm in adults, with a high metastatic rate and limited treatment approaches for systemic disease.^1^^,^^2^ UM has a distinct molecular profile, characterized by initiating mutations in the genes GNAQ and GNA11, leading to downstream activation of oncogenic pathways MAPK, PI3K/Akt, and YAP.^3^^,^^4^ At the chromosomal level, monosomy 3 (M3) is found in approximately half of all cases and is associated with a higher metastatic risk and a poorer prognosis.^5^ In addition, closely linked to M3, inactivating mutations in the tumor suppressor gene BAP1 are considered genetic drivers of metastasis development in UM.^6^^,^^7^ In contrast, variants of SF3B1 and EIF1AX are associated with a more favorable outcome.^8^^,^^9^

Although the genetics of UM has been widely explored, studies on epigenetic features remain limited.^10^^–^^15^ Epigenetic alterations, including DNA methylation, histone posttranslational modification (HPTM), and microRNA dysregulation, affect gene expression without changing the DNA sequence. Their importance is widely recognized in different types of cancer, as they have been proven to play an important role in the evolution of various neoplasms, serve as prognostic biomarkers,^16^ and represent a potential therapeutic strategy, as epigenetic traits are modifiable by specific treatments.^17^ In a previous study, we showed that specific HPTM UM patterns differed from healthy choroid and were associated with a higher tumor stage, highlighting the potential value of histone alterations in tumor prognostication.^11^ These features could also be influenced or even reversed by epigenetic drugs.^18^

However, progress in translational research has been hindered by the lack of an experimental model that accurately recapitulates the biology of this tumor.^19^ Established cell lines, though extensively explored, tend to overestimate the tumor's response to therapies. They also fail to represent UM's epigenetic landscape.^11^ Lately, patient-derived organoids (PDOs) have emerged as a promising tool for modeling various neoplasms, including UM.^19^^–^^21^

In our study, we explored an air–liquid interface (ALI) cell culture system to establish a PDO model of UM. ALI PDO represents an innovative platform for replicating key tumor characteristics, already established for other tumor types, allowing for molecular investigation and therapy testing.^22^^–^^27^ Herein, our findings support the ALI PDO model as a promising platform for future genetic, epigenetic, and therapeutic studies in UM research.

## Methods

### Uveal Melanoma Samples

Tumor tissue was obtained from five patients clinically diagnosed with UM who underwent enucleation between 2024 and 2025 at the University Eye Hospital Bonn or the University Hospital Tübingen. Patients’ demographic and clinical tumor characteristics were noted (Table 1). Patients’ consent was obtained prior to enucleation. The research was conducted in adherence to the tenets of the Declaration of Helsinki. Ethics board approval of the University of Bonn (No. 389/23) and Tübingen (346-25) was obtained. In each case, the eye was sectioned in the operating room immediately after enucleation by an ophthalmic pathologist, and tumor samples were taken for primary cell culture and for molecular testing without affecting histopathologic evaluation (for more details, see the Supplementary Material).

**Table 1.** Clinical and Histologic Characteristics of Patients

Case	Age, Y	Gender	Primary Tumor Site	Surgical Specimen	Tissue Condition	T Stage	Cell Type
UKB01	91	F	Ciliary body	Enucleation	Fresh and cryopreserved	T4d	Epithelioid
UKB02	77	M	Ciliary body	Enucleation	Fresh and cryopreserved	T1b	Spindle
UKB03	79	M	Choroid	Enucleation	Fresh and cryopreserved	T3a	Epithelioid
UKB04	58	M	Ciliary body	Biopsy and enucleation	Fresh and cryopreserved	T2b	Mixed
UKT01	91	F	Choroid	Enucleation	Cryopreserved	T3a	Mixed

### Culture of Air–Liquid Interface Patient-Derived Organoids

For cultivation of ALI PDOs, the protocol by Neal et al.^22^ was adapted. UM samples were minced and enzymatically digested in Advanced DMEM/F12 with Collagenase Type II and DNase I at 37°C for 30 to 60 minutes. After termination of digestion with fetal calf serum and subsequent centrifugation steps, the resulting cell pellet was resuspended in a mixture of culture medium and Type I-A collagen solution. This cell–matrix suspension was transferred onto 0.4-µm-pore-size Transwell inserts in a 6- or 12-well plate configuration. After a 30-minute incubation at 37°C to allow for matrix solidification, medium was added to the outer chamber. Cultures were maintained at 37°C with 5% CO_2_, with medium changes every 3 to 4 days for up to 83 days. For a detailed list of reagents, please refer to the Supplementary Material.

### DNA Sequencing

Targeted next-generation sequencing (NGS) of DNA from tumor tissue and corresponding PDO samples was performed. DNA was extracted using the Maxwell RSC DNA FFPE Kit (Promega, Madison, WI, USA) for formalin-fixed, paraffin-embedded (FFPE) tissue or the DNeasy Blood & Tissue Kit (QIAGEN, Hilden, Germany) for cell pellets. Libraries were generated with a QiaSeq custom panel (Qiagen) and sequenced on an Illumina MiSeq platform (Illumina, San Diego, CA, USA); for more details, see the Supplementary Material).

### RNA Sequencing

RNA was extracted from tumors and ALI PDOs using the RNeasy FFPE Kit (Qiagen) for FFPE sections or the RNeasy Mini Kit (Qiagen) for cryopreserved samples. Libraries were prepared using QuantSeq 3′ mRNA-Seq (Lexogen, Vienna, Austria) or Watchmaker RNA (Watchmaker Genomics, Boulder, CO, USA) kits and sequenced on an Illumina NovaSeq 6000 platform (for more details, see the Supplementary Material).

Gene Ontology (GO) overrepresentation analysis was performed using the PANTHER classification system (Overrepresentation Test, released August 7, 2024; GO Biological Process Complete annotation, GO Ontology database, released October 5, 2025). Differentially expressed genes were classified as upregulated or downregulated according to fold change direction. The reference background consisted of the 16,657 expressed genes identified in the dataset. Fisher's exact test with false discovery rate (FDR) correction was applied. GO biological processes with FDR <0.05 were considered statistically significant.^28^^–^^30^

### Quantitative Histone Posttranslational Modification Analysis by Mass Spectrometry

HPTMs were quantified in primary UM tissues and matched PDO samples using established protocols (for more details, see the Supplementary Material).^31^^–^^33^ The mass spectrometry (MS) proteomics data have been deposited in the ProteomeXchange Consortium (http://proteomecentral.proteomexchange.org) via the PRIDE partner repository^34^ with the dataset identifier PXD079705.

### Drug Screening

The ALI PDOs were cultivated in culture media until PDO growth was significant but stable, which happened between 14 and 30 days of culture. In this experimental window, we supplemented histone deacetylase inhibitor (HDACi) quisinostat (Selleck Chemicals, Houston, TX, USA. Catalog no. S10969) to the culture media to assess its effect on ALI PDOs. Each insert was treated with quisinostat at 100 nM or 200 nM (diluted in 0.3% DMSO), DMSO at 0.3%, or culture media. The wells were photographed before and after drug testing. Brightfield images were acquired using an Olympus CKX41 inverted microscope with a 4× and 10× objective (Olympus, Tokyo, Japan), with a digital camera attached. The culture plates were incubated for 72 hours, and after that, the matrix containing the PDOs was harvested.

One insert was used for the cell viability luminescence assay, performed with Cell Titer Glo 3D (Promega) and read on a SynergyH1 Microplate Reader (BioTek, Winooski, VT, USA) according to the manufacturer’s instructions. Luminescence data acquisition and analysis were performed using the Gen5 Microplate Reader Software (BioTek), and the data were processed in R (version 4.5.3) to generate dose–response graphs. The content of the remaining inserts was harvested and used for gene expression profiling and MS for HPTM analysis.

## Results

### Establishment of the UM ALI PDO Culture

Using the ALI PDO method, we successfully cultivated PDOs from five UM cases. Our ALI PDOs were successfully established from fresh tissue for four patients and from cryopreserved tissue for all five patients. PDO growth increased substantially between 2 and 4 weeks of culture, stabilized between 4 and 8 weeks, and declined progressively after 8 weeks. Cultures of different tumors displayed variable growth rates, with some forming PDOs within 7 days of incubation, while others required up to 14 days. Passaging was attempted after 1 month in culture with 1:2 to 1:3 split ratios, but culture growth after splitting was slow, with progressive degeneration after 1 week. The PDOs did not survive after 11 weeks of culture. Signs of devitalization of culture included progressive darkening of the PDO center, disintegrating or shrinking structures, and consistently yellowish media. Microscopically, the PDOs were variably pigmented cellular masses, showed morphologic similarities to the primary tumors, and maintained positivity for melanocyte immunomarkers MART-1, HMB-45, and SOX10, replicating the morphology and immunophenotype of their corresponding primary tumors (Fig. 1). Lymphocytes and macrophages were not present at different culture time points (earliest assessment at D6) by hematoxylin and eosin and immunohistochemical stains for CD68 and CD8. However, the original tumor FFPE sections displayed variable immune cell infiltration by immunohistochemistry.

**Figure 1.**
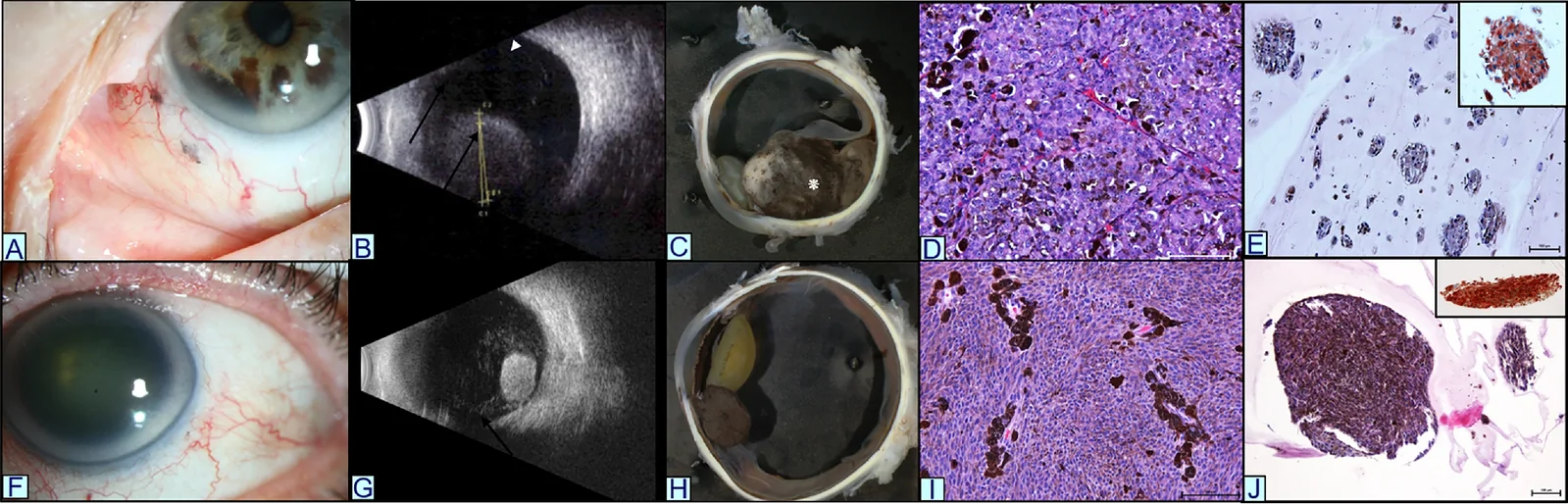
Clinical and histologic correlation of (**A–E**) UKB01 and (**F–J**) UKB02. (**A**) Ciliary body melanoma infiltrating the iris and extraocular extension. (**B**) Ultrasound image. (**C**) Macroscopic view after sectioning. The tumor is mainly pigmented. (**D**) The tumor is composed of epithelioid cells intermingled with pigmented macrophages (hematoxylin and eosin [H&E] stain, *scale bar* = 100 µm). (**E**) Corresponding ALI PDO. The *inset* shows strong positivity of ALI PDOs for the marker MART-1, which confirms melanocytic lineage (H&E stain, *scale bar* = 100 µm). (**F**) Clinical picture of the eye showing only tiny episcleral pigmentation. (**G**) Ultrasound image and (**H**) macroscopic picture after sectioning showing a ciliary body tumor. (**I**) The tumor is mainly composed of spindle cells with pigment-laden macrophages (H&E stain, *scale bar* = 100 µm). (**J**) Corresponding ALI PDO (H&E stain, *scale bar* = 100 µm), with *inset* showing positivity for the marker MART-1.

### DNA Sequencing Confirms Molecular Fidelity of ALI PDOs and Tumor of Origin

To assess the genetic stability of ALI PDOs relative to their tumors of origin, targeted DNA sequencing was performed on matched tumor tissues and derived PDOs. Overall, tumor-specific variants were largely preserved in the corresponding ALI PDOs, supporting the close molecular relationship between the models and their tissue of origin. Notably, in our third case, EIF1AX appeared homozygous in the PDOs (allele frequency of 100%). This finding might reflect loss of heterozygosity and/or the enrichment of pure tumor cells in culture compared with the heterogeneous cellular composition of the bulk tumor tissue. However, potential clonal selection during culture expansion cannot be excluded.

Across all samples, driver mutations were consistently retained, with minor variations in allele frequencies likely attributable to clonal selection or technical differences in sequencing depth (Table 2). Variants classified as likely oncogenic or pathogenic in ClinVar and OncoKB were reliably observed in PDOs, indicating preservation of the tumors’ key oncogenic features. Collectively, these findings demonstrate that ALI PDOs faithfully recapitulate the genetic landscape of their primary tumors.

**Table 2.** Genetic Profile of ALI PDOs and Corresponding Primary Tumors

Sample Name	Gene	HGVSc	HGVSp	AF %
UKB01 Tumor	BAP1	c.2015A>G	p.Asp672Gly	82.14
	GNA11	c.626A>T	p.Gln209Leu	50.00
UKB01 ALI PDO	BAP1	c.2015A>G	p.Asp672Gly	100.00
	GNA11	c.626A>T	p.Gln209Leu	77.78
UKB02 Tumor	BAP1	c.836C>G	p.Ser279	87.76
	GNA11	c.626A>T	p.Gln209Leu	34.09
UKB02 ALI PDO	BAP1	c.836C>G	p.Ser279	90.48
	GNA11	c.626A>T	p.Gln209Leu	33.33
UKB03 Tumor	GNAQ	c.626A>C	p.Gln209Pro	45.71
	EIF1AX	c.25_30del	p.Gly9_Lys10del	88.23
UKB03 ALI PDO	GNAQ	c.626A>C	p.Gln209Pro	51.11
	EIF1AX	c.25_30del	p.Gly9_Lys10del	100.00
UKB04 Tumor	BAP1	c.535C>T	p.Arg179Trp	93.04
	GNAQ	c.626A>T	p.Gln209Leu	40.16
UKB04 ALI PDO	BAP1	c.535C>T	p.Arg179Trp	80.36
	GNAQ	c.626A>T	p.Gln209Leu	27.66
UKT01 Tumor	BAP1	c.665_666delCC	p.Pro222Leufs*20	76.43
	GNA11	c.626A>T	p.Gln209Leu	44.50
UKT01 ALI PDO	BAP1	c.665_666delCC	p.Pro222Leufs*20	97.27
	GNA11	c.626A>T	p.Gln209Leu	51.98

HGVSc indicates coding DNA change; HGVSp indicates protein change. AF%, allele frequency (%); HGVS, Human Genome Variation Society.

### Gene Expression Analysis Shows Similarities Between Primary Tumor and Respective PDOs

RNA sequencing (RNA-seq) analysis was used to compare the gene expression profiles of ALI PDOs with their matching tumors. The results showed that only 494 of 16,657 genes were differentially expressed at FDR <0.05 between ALI PDOs and their matched tumors (Fig. 2A). Of these 494 differentially expressed genes, 406 were upregulated and 88 were downregulated in UM tissue compared with the ALI PDO. A volcano plot highlights the differentially expressed genes, which mainly account for immune- and vascular-related signals (Fig. 2A). GO overrepresentation analysis using the PANTHER classification system identified strong enrichment for immune- and microenvironment-associated processes among genes upregulated in tumor tissue compared with PDOs. The significantly enriched categories included immune response, antigen processing and presentation via major histocompatibility complex class II, complement activation, B-cell–mediated immunity, and angiogenesis-related processes (FDR <0.05). Such findings are consistent with the absence of stromal components in the PDOs. Conversely, no GO biological processes were significantly enriched among downregulated genes after multiple testing correction (Fig. 2B). The principal component analysis (PCA) plot shows that ALI PDOs and their original tumors clustered together (Fig. 2C). The primary tumor samples also tended to cluster on their own (Fig. 2D), as did the quisinostat-treated ALI PDO compared to the controls (DMSO/media).

**Figure 2.**
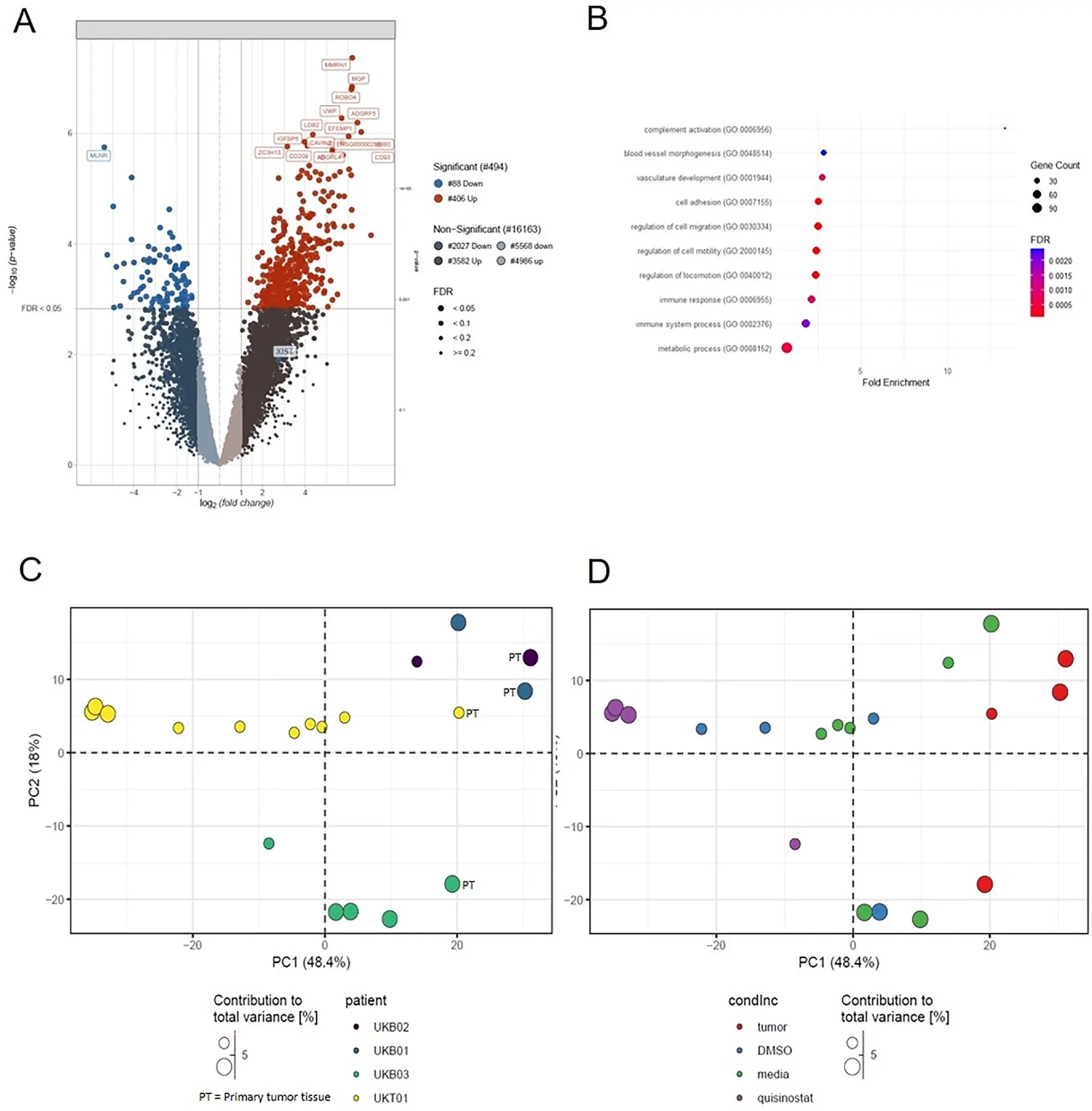
Gene expression. (**A**) Volcano plot highlighting the differentially expressed genes. The top upregulated genes in primary tumor samples reveal a predominant vascular/endothelial- and immune-related signal. (**B**) Overall, those genes are associated with vascular and immune processes, including angiogenesis and endothelial adhesion in the gene ontology analysis. (**C**) PCA plots show that samples of tumor tissue and respective ALI PDOs tend to cluster together according to each case. (**D**) The primary tumor samples also tend to cluster on their own, as did the ALI PDOs (in medium and DMSO) and the quisinostat-treated cells.

Overall, given the low number of differentially expressed genes and the clustering of PDOs and their matched tumor samples in PCA, we conclude that the gene expression profiles of ALI PDOs and their matched tumors remain very similar (except for immune- and microenvironment-associated processes), supporting their close molecular resemblance.

### ALI PDOs Recapitulate the HPTM Landscape of Primary Tumors

To comprehensively characterize the epigenetic landscape of uveal melanoma ALI PDOs, we profiled HPTMs using an MS-based approach. This method enables unbiased, quantitative analysis across multiple residues and overcomes the specificity and multiplexing limitations of antibody-based methods.

Four primary UM tissues and matching ALI PDOs were compared (Fig. 3). Two PDO samples obtained at different time points were analyzed for sample UKB01-03. The amount of material obtained from ALI PDOs was sufficient to profile the most common acetylations and methylations on histones H3 and H4, with the exception of one sample, for which a more limited set of peptides could be quantified. The comparison of primary UM tissues with their matched PDOs revealed reproducible differences in specific HPTMs (Figs. 3A, 3B). In particular, several acetylated histone H3 and H4 peptides—including combinatorial marks such as H3K18ac│K23ac and H3K9acK14ac, as well as acetylated histone H4 species—were consistently reduced in PDOs relative to the corresponding tumor tissues. The observed global reduction in histone acetylation in PDOs likely reflects adaptive changes induced by in vitro culture conditions, such as altered metabolic inputs and absence of microenvironmental signals, which are known to influence histone acetylation dynamics.^35^

**Figure 3.**
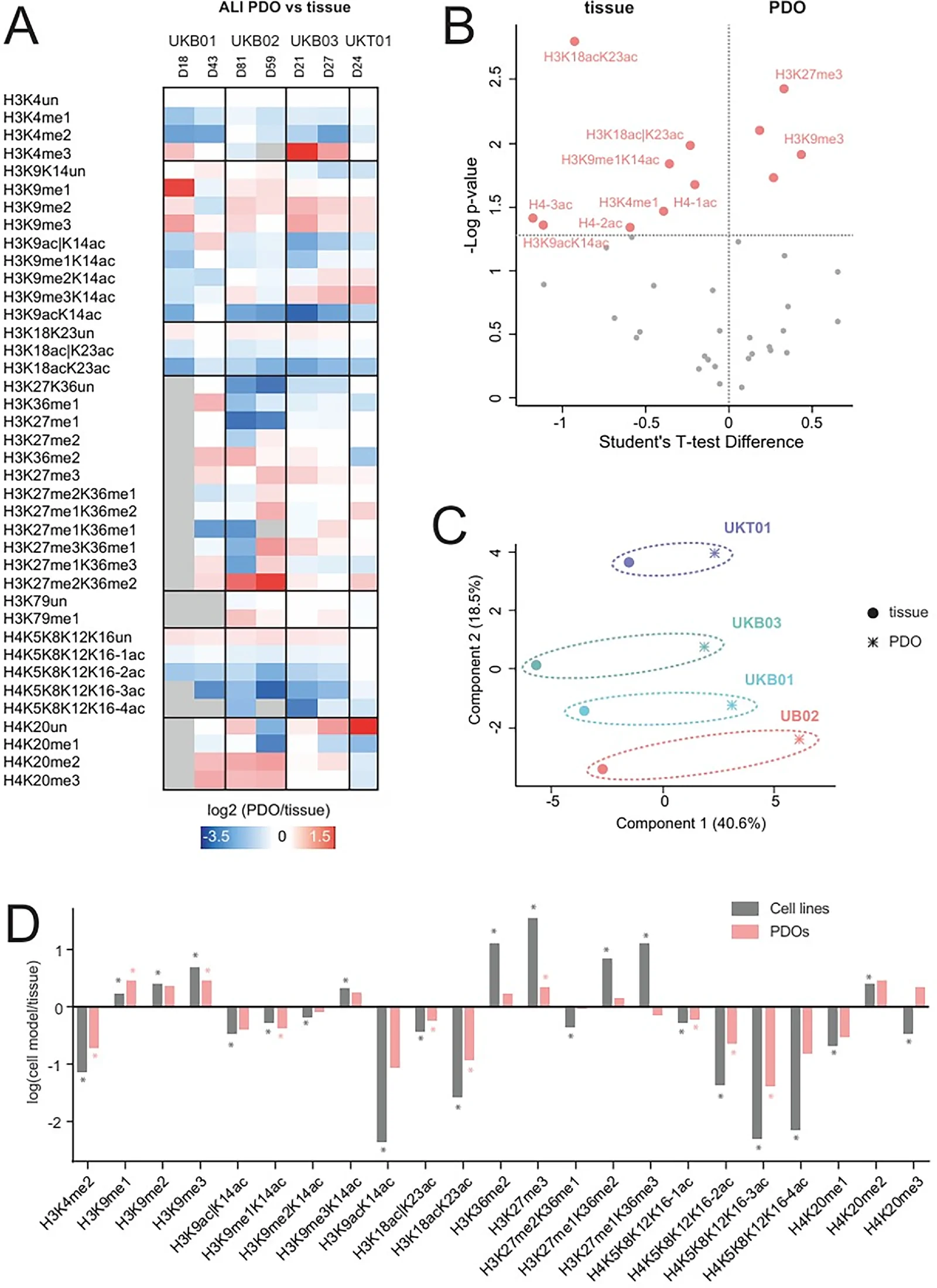
Analysis of posttranslational histone modifications. MS-based comparison of HPTM profiles between primary uveal melanoma tumors and matched ALI PDOs. (**A**) Heatmap showing the log2 fold change (PDO/tissue) of differentially modified histone peptides. (**B**) Volcano plot showing the Student's *t*-test difference versus statistical significance (−log *P* value) for individual HPTMs in PDOs compared to tissues. (**C**) PCA of global HPTM profiles, showing clustering of primary tumors (*dots*) and matched PDOs (*asterisks*), indicating preservation of patient-specific epigenetic signatures. Averages were used for samples from which two ALI PDO samples were analyzed. (**D**) Comparison of log fold changes (model/tissue) for individual histone PTMs between ALI PDOs and established UM cell lines, based on a previously published dataset.^11^ Only peptides showing a significant difference between cell lines and tissues are shown. *Gray* *asterisks*: *P* < 0.05 by unpaired two-tailed Student's *t*-test between 24 UM tissues and 16 cell lines. *Red asterisks*: *P* < 0.05 by paired two-tailed Student's *t*-test between 4 UM tissues and matched PDOs.

In contrast, PDOs showed increased repressive histone methylation marks, most notably H3K27me3 and H3K9me3, along with increased mono-acetylated H4 (H4-1ac).

Despite these differences, a PCA demonstrated that PDOs largely retained patient-specific clustering patterns relative to their tumors of origin (Fig. 3C), indicating preservation of intersample epigenetic heterogeneity. These results support the notion that ALI PDOs recapitulate key epigenetic features of the primary UM tumors, despite culture-associated shifts in specific chromatin marks.

To further assess how ALI PDOs recapitulate the epigenetic landscape of primary UM tumors, we compared the HPTM changes in PDOs with those previously observed in UM cell lines (Fig. 3D).^11^ Most histone modifications significantly altered in PDOs follow the same trend seen in cell lines compared to tumor tissues. However, changes are consistently less pronounced in PDOs, as shown by lower absolute log fold change values. These results indicate that ALI PDOs preserve histone modification patterns more faithfully than conventional cell lines. This supports their use as a more representative ex vivo model of the tumor epigenetic landscape.

### ALI PDO Is a Useful Platform for Drug Testing

To evaluate epigenetic drug responsiveness, PDOs from three cases (UKB01, UKB03, and UKT01) were incubated for 72 hours with the HDACi quisinostat (100 nM and 200 nM), with matched media and DMSO samples serving as controls. Quisinostat was selected based on its recent clinical relevance, as it is under investigation in a phase 2 adjuvant clinical trial for high-risk UM (NCT06932757).

In the morphologic assessment under brightfield imaging, no substantial difference was observed between treated and untreated ALI PDOs. A qualitative analysis comparing photographs of treated and untreated wells showed no evident morphologic differences between groups. However, in a luminescence-based cell viability assay, signals varied significantly across the five culture cases, with a reduction in cell viability in three of four PDOs, reflecting case-dependent response variability (Fig. 4A).

**Figure 4.**
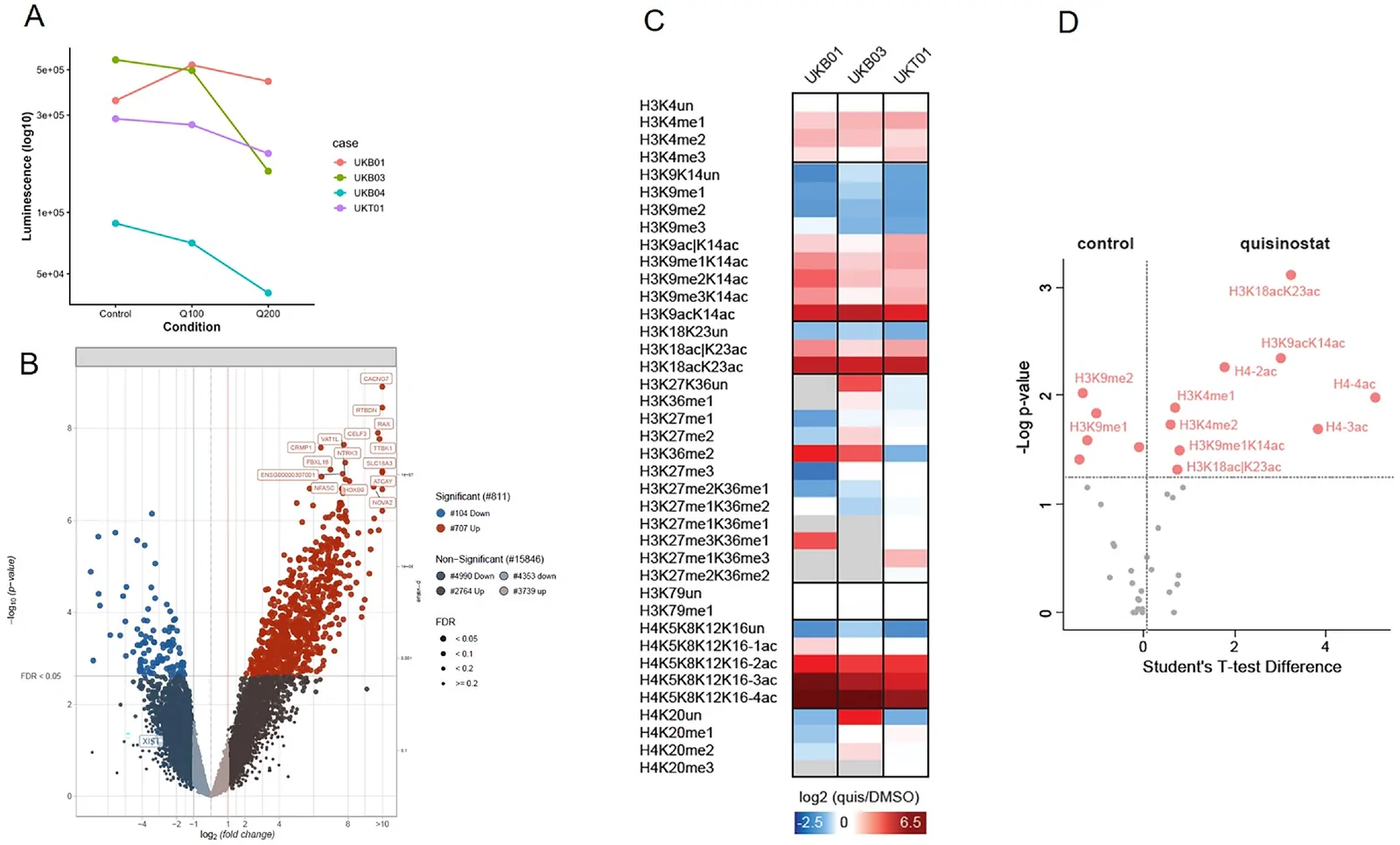
(**A**) Posttreatment changes: dose–response curve of UM ALI PDOs used for drug screening. Each line represents a different ALI PDO culture, with points indicating median values per condition (control, quisinostat 100 nM, and quisinostat 200 nM) on a logarithmic scale. (**B**) Differential gene expression induced by HDACi treatment in UM ALI PDOs. The volcano plot shows differential gene expression between quisinostat-treated and control PDOs (based on two tumors and eight ALI PDO samples). Significantly upregulated genes are shown in *red* and downregulated genes in *blue* (FDR <0.05). HPTM changes in ALI PDOs induced by HDACi. (**C**) Heatmap showing the log2 fold change between quisinostat–treated PDOs and untreated controls. Treatment resulted in a marked increase in several histone acetylation marks, consistent with inhibition of histone deacetylases. (**D**) Plot shows the Student's *t*-test for individual histone marks, illustrating differences between treated and control PDOs.

### Gene Expression in the ALI PDOs Is Affected by HDAC Inhibition

Gene expression profiling by RNA-seq identified 811 differentially expressed genes (FDR <0.05), of which 707 were upregulated and 104 downregulated in the treated group. Among the most induced genes are CACNG7, RTBDN, RAX, TTBK1, SLC18A3, and NOVA2, associated with neural/neuroectodermal programs. Meanwhile, some genes related to cell proliferation and melanocytic differentiation were underrepresented, such as RRM2, KIF20A, MLANA, and SLC24A5 (Fig. 4B). The downregulation of RRM2, involved in DNA synthesis, might have a relevant antiproliferative effect.^36^ Overall, the results suggest a broad transcriptional reprogramming induced by HDAC inhibition.

### Treatment of ALI PDOs With HDACi Had the Expected Epigenetic Response

Upon treatment with the HDAC inhibitor quisinostat, several increased acetylation marks were observed (Fig. 4C). There was a consistent increase in H3 acetylation, particularly in H3K18ac, H3K18ac│K23ac, H3K9acK14ac, H3K9me2K14ac, and H3K9me3K14ac. Modifications in H4 were also noticed, with upregulation of multiacetylated forms at K5, K8, K12, and K16 (2ac, 3ac, and 4ac) (Fig. 4D). These changes were consistent across PDO samples and aligned with the expected biochemical effects of HDAC inhibition. Together, these results suggest that the ALI PDOs preserve tumor-specific histone profiles and respond to epigenetic-modifying therapy.

## Discussion

Our study showed that the ALI PDO method allows for a successful propagation of primary UM tissue, both fresh and cryopreserved, and is maintainable over a suitable experimental window. The PDOs retained characteristics similar to their original tumors regarding histologic, immunohistochemical, genetic, and epigenetic features, as well as their molecular profile. They also reported personalized responses to treatment with the HDACi quisinostat, demonstrating that the system can be influenced as expected by external modifiers. Altogether, these findings position the ALI PDO model as a promising platform for further translational research in UM.

UM is an entity with molecular aspects that differ considerably from other types of melanoma, including a restricted mutational profile, low mutational burden, and a less explored epigenetic landscape. Current in vivo and in vitro models for studying UM have limited translational value. Established cell lines, though widely used, tend to overestimate therapeutic responses and do not reflect the tumor's epigenetic aspects well.^11^^,^^37^ They also do not offer the possibility of assessing individual differences in the effect of interventions. In contrast, animal models, including patient-derived xenografts (PDXs), though more representative of a real organism's response to tumor development and treatment, are technically complex and costly.^38^ They are also limited regarding aspects of the tumor microenvironment as the mice are immunocompromised. In this context, PDO models arise as an intermediate alternative with great potential, allowing for the preservation of tumor features during propagation while having the advantage of controlled cell manipulation.^20^^,^^21^ A previous study created a biobank of UM PDOs, cultivated within a Cultrex matrix, using a conventional submerged culture setting. However, they transitioned to a PDX setting for therapy testing.^19^ Although this has the above-mentioned limitations, it has been shown for other tumor entities that cell culture–induced HPTM changes may be partially reversed to the in vivo situation using PDX models.^35^

The ALI PDO model was established by Neal et al.^22^ for multiple human tumor types and later used by other groups in translational oncology research. Our work, to the best of our knowledge, is the first to successfully apply the method to UM tissue culture for tumor modeling and ex vivo targeted drug screening. The retention of UM driver mutations sustains the integrity of the model and is clinically relevant, as several genetic alterations have been shown to define prognostically different molecular subgroups. Interestingly, the small differences in allele frequencies of genetic variants between primary tumor and PDO samples suggest clonal selection during ex vivo cell culture. This phenomenon has been observed in cell cultures of other tumor types, underscoring the importance of periodic monitoring of genomic stability throughout culture to ensure cell populations remain representative of their original tissue.

Furthermore, RNA-seq analysis revealed mostly similarity between UM ALI PDOs and their original tumors. Only 494 genes were significantly differentially expressed among the 16,657 genes analyzed. GO analysis showed that tumor cell–intrinsic programs were preserved, with the main differences involving processes related to vascular and immune components, which were absent in the cultures. Such a pattern was also observed by Esser et al.,^23^ who found that their ALI PDOs of renal cell carcinoma and primary tumors differed in gene expression linked to vascular and immune signals. Our results support our hypothesis that the ALI PDO system reflects the overall gene expression profile of UM.

Another important aspect of our study is its focus on the HPTM of tumors and their PDOs, an epigenetic feature that is still little explored in UM. However, HPTM has been found to be of significant diagnostic and also therapeutic value in other types of cancer.^16^ Using an MS-based analysis, we previously obtained an accurate quantitative view of the histone landscape of UM tissues and cell lines.^16^^,^^33^^,^^39^ In the data from that study, UM cell lines displayed marked alterations in histone PTM profiles compared to primary tumors, consistent with previous studies analyzing the effect of culture conditions on epigenetic patterns in multiple cellular models,^35^ indicating a substantial epigenetic divergence associated with long-term in vitro adaptation.^11^ Here, we add another layer of information by comparing ALI PDOs with their matched tissues. While PDOs showed several HPTM changes that occurred in the same direction as those observed in UM cell lines, these changes were markedly less pronounced. In addition, despite these differences, the overall patient HPTM landscape remained preserved, and the PDOs mirrored patient-specific epigenetic characteristics. These results further support ALI PDOs as models for studying chromatin regulation in UM, as well as their potential utility to investigate the prognostic value of these epigenetic marks in a controlled experimental context.

Although previous studies, including Dalvin et al.,^19^ have demonstrated the feasibility of drug testing in UM PDOs, our study extends the characterization of these models by integrating quantitative HPTM profiling using MS. Unlike chromatin accessibility assays, MS directly quantifies multiple HPTMs and their combinatorial states, providing an additional molecular dimension for evaluating the epigenetic landscape of UM PDOs. Importantly, because quisinostat directly targets HDACs, the primary molecular consequence of treatment is a change in histone acetylation. MS therefore provides a direct and quantitative readout of target engagement by measuring histone acetylation levels, whereas chromatin accessibility assays capture downstream changes in chromatin organization rather than the biochemical modifications themselves.

Regarding the applicability of the ALI PDOs for drug screening, our tests with quisinostat showed that the PDOs are susceptible to epigenetic modulation, having displayed a consistent increase in multiple acetylation marks in histones H3 and H4, which is in line with the expected mechanism of action of an HDACi.^40^^–^^43^ In parallel, gene expression analysis after treatment identified downregulation of genes associated with cell proliferation programs and melanocytic identity, such as RRM2, KIF20A, MLANA, and SLC24A5. The downregulation of RRM2, which encodes a ribonucleotide reductase subunit essential for DNA synthesis, is particularly relevant from an antiproliferative standpoint.^36^^,^^44^ Taken together, these findings suggest that HDAC inhibition induced broad transcriptional reprogramming in UM ALI PDOs, with possible redirection toward a less proliferative phenotype. Furthermore, the variable therapeutic response among cases, observed in the cell viability assay and molecular profiles, reflects the biological heterogeneity among patients and reinforces the value of the ALI PDO model for precision medicine strategies. Future research may focus on more selective HDACis to further clarify the specificity of the ALI PDO drug response.

Our study, however, presents limitations that must be acknowledged. The number of cases is restricted, which is inherent to the pilot nature of the work and the rarity of this neoplasm. Also, the longevity of our PDOs was limited to approximately 11 weeks, with a progressive decline in viability after the eighth week of culture, and passaging did not result in sustained growth. This limitation contrasts with results from previous research that reported more passages using different culture systems.^19^ However, since our aim is to have a model that recapitulates the primary UM as best as possible, the failure of passaging may not be a major limitation. As passaging may result in molecular changes of the PDOs, it might reduce their representativeness. Therefore, the narrow experimental window of our ALI PDO model might also indicate its closer resemblance to the biology of the primary tumor.

Furthermore, the absence of an intervening vascular and immune component hinders the replication of the tumor microenvironment. In previous studies, the ALI culture system allowed the preservation of elements of the tumor stroma, including fibroblasts and immune populations, more effectively than other culture systems.^22^^,^^23^ However, in the present study, lymphocytes and macrophages or a significant stromal component were not detected by immunohistochemistry in PDO samples analyzed at different culture points. This finding may reflect the early loss of immune cells during culture and is consistent with our transcriptomic data. Future perspectives include expanding the cohort to encompass a greater diversity of molecular profiles and improving culture strategies for reconstituting the immune microenvironment.

Additionally, while MS provided a global quantitative view of the HPTM landscape, our study did not include gene-specific epigenetic mapping (e.g., Chromatin ImmunoPrecipitation DNA-Sequencing), which could directly correlate the level of histone marks with gene expression. Future studies incorporating gene-specific epigenomic approaches will be important to further dissect the molecular mechanisms underlying the transcriptional changes identified in this work.

## Conclusions

This pilot study demonstrated that the ALI culture system is a viable and effective platform for establishing UM PDOs, which recapitulate the overall genetic, epigenetic, and molecular profile of the primary tumors. In particular, the preservation of HPTM signatures highlights the value of this model for future studies on chromatin regulation in UM. Furthermore, the functional response and transcriptional reprogramming observed after HDAC inhibition validate the platform's potential for ex vivo drug screening and the development of personalized therapeutic approaches. Despite some limitations to overcome, the ALI PDO model establishes itself as a valuable translational resource, filling the gap of conventional experimental models.

## Supplementary Material

Supplement 1
