# Supplementary material for: Establishment of Uveal Melanoma Patient-Derived Organoids Using an Air–Liquid Interface System for Translational Research
Source: Invest Ophthalmol Vis Sci. 2026 Oct 1;67(12):1. doi: 10.1167/iovs.67.12.1 (PMC13648566; doi:10.1167/iovs.67.12.1)
Supplement: Supplementary file 1 [file iovs-67-12-1_s001.docx]

**Online only / Supplementary material**

**Methods**

***Uveal Melanoma Samples***

Tumor tissue was obtained from five patients clinically diagnosed with UM, who underwent enucleation between 2024 and 2025 at the University Eye Hospital Bonn or at the University Hospital Tübingen. Patients’ demographic and clinical tumor characteristics were noted (Tab. 1) The patients’ consent was obtained prior to enucleation. The research was conducted in adherence to the tenets of the Declaration of Helsinki. Ethic Board Approval of the University of Bonn (No. 389/23) and Tübingen (346-25) was obtained. In each case, the eye was sectioned in the operating room immediately after enucleation by an ophthalmic pathologist and tumor samples were taken for primary cell culture and for molecular testing without affecting histopathological evaluation. After sampling, the enucleated eye was fixed in 4% paraformaldehyde for 48 hours and processed and reported according to current guidelines. In brief, Hematoxylin-Eosin (H&E) and Periodic Acid-Schiff (PAS) staining were performed on 5 μm-thick sections from formalin-fixed, paraffin-embedded (FFPE) blocks. Immunohistochemical stains for MART-1 (clone A19-P, Novus Biologicals, NBP1-30151; 1:400), HMB-45 (clone HMB-45, Dako, M0634; 1:100), SOX10 (clone A-2, Santa Cruz Biotechnology, sc-365692; 1:100), CD68 (clone PG-M1, Dako, M0876; 1:100), and CD8 (C8/144B, Invitrogen; 1:30) were performed for subsequent pathological evaluation of each tumor. The diagnosis of uveal melanoma was confirmed, and the histopathological T Stage was reported according to the TNM AJCC cancer (8th edition).

***Cryopreservation of Tumor Samples for PDO Culture***

We seeked to preserve tumor fragments for posterior culture by storing them in cryovials containing 1 mL of CryoStor CS10 (BioLife Solutions). The vials were placed in a CoolCell freezing container (Corning) to ensure a cooling rate of approximately -1 °C/minute and kept at -80 °C for at least 4 hours. Subsequently, the specimens were transferred to ultra-low temperature storage (-150 °C) for long-term preservation.

***Culture of Air-Liquid Interface Patient-Derived Organoids***

For cultivation of ALI PDO the protocol by Neal *et al* (2018) was applied. A 6- or 12-well plate was previously prepared with inserts 0.4 μm pore size Transwell® inserts and a layer of collagen mixture. For that, Collagen Matrix (Cell Matrix Type I-A, Fujifilm Wako Chemicals Europe) was mixed with 10x concentrated Ham's F12 (Ham's F-12 Nutrient Mix, Thermo Scientific) supplemented with Normocin (Invivogen) and Reconstitution Buffer (2.2 g NaHCO3 in 100 ml 0.05 N NaOH and 200 mM Hepes) in a ratio of 8:1:1. The prepared solution was used to coat each insert and was reserved for embedding the tumor cells to be cultured. Subsequently, UM samples were minced into millimetric pieces through pipetting and carefully digested in Advanced DMEM/F12 (Thermo Fisher Scientific) supplemented with GlutaMAX (Thermo Fisher Scientific), Penicillin/Streptomycin (Thermo Fisher Scientific), HEPES (Thermo Fisher Scientific), Collagenase Type II (Sigma-Aldrich), DNase I (Sigma-Aldrich), and Y-27632 (Abcam). The suspension was incubated in a 37°C water bath for 30 to 60 minutes and the extent of diges-tion was monitored every 15 minutes by light microscopy. To terminate enzymatic digestion, fetal calf serum (Sigma-Aldrich) was added, as well as supplemented AdDMEM/F12. The suspension was centrifuged at 300×g for 5 minutes at 8°C. The supernatant was carefully aspirated, and the pellet was resuspended in supplemented AdDMEM/F12 and centrifuged again under the same conditions. The resulting pellet was finally resuspended in 25–50 μL of a mixture composed of culture medium and collagen solution. This cell–matrix suspension was transferred into Transwell® insert (pre-coated with Type I-A collagen), placed in plate, and incubated at 37°C for 30 minutes. Subsequently, culture medium was added to the outer chamber between insert and well. The plates were incubated at 37°C with 5% CO2. Culture medium (see Supplement Table 1) was replaced every 3–4 days. Prior to each medium change, PDOs were inspected and imaged using an inverted phase-contrast microscope (Olympus CKX41). The ALI PDO were cultivated for up to 83 days.

*Supplement Table 1: Composition of ALI culture media:*

Cell viability was routinely monitored qualitatively via inverted light microscopy every 3-4

days during media changes. Parameters included: PDO size and number; signs of culture devitalization (such as progressive central darkening, disintegration, or shrinking of PDOs). In cases where morphological assessment was inconclusive, live/dead fluorescent staining with Calcein-AM (Thermo Fisher Scientific) was performed to confirm viability (Supplement Figure 1). For quantitative viability assessment during drug screening, the CellTiter-Glo 3D CellViability Assay (Promega) was used. Luminescence was measured on a SynergyH1 Microplate Reader (BioTek) using Gen5 Microplate Reader Software (BioTEk) according othe manufacturer’s instructions.

Supplement Figure 1: Assessment of cell viability in ALI PDOs. On day 44, brightfield imaging raised doubts about the vitality of the UKB01 culture (A). Calcein-AM fluorescent staining was performed to confirm cell viability (B).

***Splitting UM ALI PDOs***

Passaging was attempted when the cultures reached confluency, the collagen matrix appeared disintegrated, or the culture media became consistently acidic (yellowish). The collagen matrix containing the PDOs was carefully mechanically detached from the Transwell® insert using a scraper or pipette tip, and transferred into a tube containing collection media (Advanced DMEM/F12 supplemented with GlutaMAX, Penicillin/Streptomycin, and HEPES), and Collagenase Type IV (10 mg/mL; STEMCELL Technologies). The suspension was incubated for 30 minutes at 37 °C and centrifuged at 400 × g for 5 minutes. After discarding the supernatant, the cell pellet was resuspended in 10 mL of collection media and centrifuged again at 400 × g for 5 minutes. Finally, the pellet was resuspended in 30 µl of culture media, mixed with a fresh collagen matrix, and placed onto pre-coated inserts at split ratios of 1:2 to 1:3. The new plate was maintained incubated as previously described.

***Histological Processing of ALI PDOs***

For histological assessment, the collagen matrix embedding the ALI PDOs was scratched from the insert and fixed in paraformaldehyde 4% solution (PFA) inside an Eppendorf tube. To prevent loss during processing, the sample was carefully wrapped in biopsy wrap paper before being placed in a tissue cassette. The material was processed through a standard dehydration protocol, cleared, and embedded in paraffin. FFPE blocks were sectioned using a microtome, and 5µm thick sections were mounted onto glass slides. Hematoxylin and eosin (H&E) and immunohistochemistry (IHC) stainings were performed according to routine staining protocols of our Ophthalmic Pathology Laboratory. For IHC, deparaffinized and rehydrated sections underwent heat-induced antigen retrieval in citrate buffer (pH 6.0) for 20 minutes. Following endogenous peroxidase blocking (3% H_2_O_2_) and non-specific site blocking (5% BSA in TBS), slides were incubated with primary antibodies overnight at 4 °C. Detection was performed using an HRP-conjugated secondary antibody and DAB chromogen, followed by Mayer's hematoxylin counterstaining and mounting. Stains used were MART-1 (clone A19-P, Novus Biologicals, NBP1-30151; 1:400), CD68 (clone PG-M1, Dako, M0876; 1:100) and CD8 (C8/144B, Invitrogen; 1:30) (Supplement Figure 2).

Supplement Figure 2: Histological and immunohistochemical characterization of primary UM tissue and corresponding PDOs. Sections were stained with HE, MART1 (to confirm melanocytic identity of the PDOs), as well as CD68 and CD8 (to assess the presence of macrophages and T lymphocytes, respectively). No CD68 or CD8 positivity was identified in the PDOs, as the brownish pigmentation observed corresponds to melanin deposition

***DNA Sequencing***

Next generation sequencing (NGS) of DNA extracted from tumor tissue and corresponding PDO samples was performed to determine UM specific mutations of GNAQ, GNA11, CYSLTR2, PLCB4, EIF1AX, SF3B1 and BAP1. DNA extraction was carried out either from FFPE tumor sections using the Maxwell® RSC DNA FFPE Kit (cat. no. AS1450; Promega Corporation, Madison, WI, USA) or from fresh-frozen ALI PDO cell pellets using the DNeasy Blood & Tissue Kit (cat. no. 69504; QIAGEN, Hilden, Germany), following the manufacturers’ protocols. DNA concentration was assessed using the QuantiFluor® ONE dsDNA System (cat. no. E4870; Promega Corporation, Madison, WI, USA).

Sequencing libraries were prepared using a QiaSeq DNA custom panel (Qiagen) with an in-put of up to 40 ng DNA, following the manufacturer’s protocol. Libraries were subjected to NGS on a MiSeq sequencing platform (Illumina). Sequencing data were processed and analyzed for genomic variants using the CLC Genomics Workbench (Qiagen). Variants were manually curated to exclude sequencing artifacts, synonymous variants, and presumed germline alterations. Only somatic variants with an alternative allele frequency ≥5% were retained. Variant classification and interpretation were carried out using dbSNP, ClinVar, On-coKB, and Mastermind Genomic Search Engine in their current versions.

***RNA Sequencing***

RNA was extracted from primary tumors and ALI PDOs at different culture time points (see Supplement Table 2). For FFPE tissue, the RNA extraction was performed using the RNeasy FFPE Kit (QIAGEN). For cryopreserved tissue, the RNeasy Mini Kit (QIAGEN) was used. RNA concentration and purity were assessed by NanoDrop spectrophotometry (Thermo Fisher Scientific). RNA-sequencing (RNA-seq) libraries were prepared using either the QuantSeq 3’ mRNA-Seq Library Prep Kit FWD (Lexogen) or the Watchmaker RNA Library Prep Kit (Watchmaker Genomics), and sequenced on an Illumina NovaSeq 6000 platform, with average sequencing depth of 10 million reads/sample and 50 million reads/sample, respectively.

The nf-core rnaseq pipeline (version 3.14.0) with default parameters was used for the preprocessing and the quantification of the reads using the human reference genome GRCh38.^45^ Differential expression analysis was performed with the Bioconductor package limma considering only genes with ≥100 total counts and a minimum of 30 counts in each of at least 2 samples.^46^ The condition (3 PDO groups or tumor) of the sample and its donor were modelled as fixed effect and replicates defined by the combination of donor, condition and sequencing batch were modelled as a random effect via the duplicateCorrelation function (limma package).^46^ Statistical contrasts were calculated between conditions and between the average of the control conditions and the tumor. The Benjamini-Hochberg method was applied to calculate multiple testing-adjusted p-values for each statistical contrast.

***Quantitative Histone Post-Translational Modification Analysis by Mass Spectrometry***

HPTMs were quantified in primary UM tissues and matched PDO samples using established protocols.^31–33^ Histones were enriched from either cryopreserved or FFPE tissue (10 sections of 10 µm) or from PDO pellets containing approximately 1 × 10^5^ cells, as previously described.^31^ Up to 4µg of histone octamer were mixed with an equal amount of heavy-isotope-labeled histones, which were used as an internal standard,^32^ and separated on a 17% SDS-PAGE gel. Histone bands were excised, chemically acylated with propionic anhydride, and in-gel digested with trypsin.^33^ The samples were separated by reversed-phase chromatography, acquired on a Q Exactive Plus instrument (Thermo Fisher Scientific), and analyzed using MaxQuant and QualBrowser.^33^ For each histone-modified peptide, the percentage relative abundance (%RA) value for the sample (light channel, L) or the internal standard (heavy channel, H) was estimated. Differential levels of individual HPTMs were assessed using a two-tailed Student’s t-test. The results were visualized using volcano plots and principal component analysis (PCA) using Perseus.^47^

Supplement Table 2: Summary of primary tumor and ALI PDO sampling timeline and analytical methods.

**References**

45.	Ewels PA, Peltzer A, Fillinger S, et al. The nf-core framework for community-curated bioinformatics pipelines. *Nat Biotechnol*. 2020;38(3):276-278.

46.	Ritchie ME, Phipson B, Wu D, et al. Limma powers differential expression analyses for RNA-sequencing and microarray studies. *Nucleic Acids Res*. 2015;43(7):e47.

47.	Tyanova S, Cox J. Perseus: a bioinformatics platform for integrative analysis of proteomics data in cancer research. *Methods Mol Biol*. 2018;1711:133-148.
